# A predictive nomogram for incomplete clinical success after unilateral adrenalectomy in patients with primary aldosteronism

**DOI:** 10.3389/fendo.2025.1628564

**Published:** 2025-07-24

**Authors:** Pin Wang, Limei Liu, Sen Lu, Xianjun Zhu, Rui Zhu, Yan Yang, Guangpeng Zhou, Xu Cao

**Affiliations:** ^1^ Department of Endocrinology, Sichuan Provincial People’s Hospital, School of Medicine, University of Electronic Science and Technology of China, Chengdu, China; ^2^ Department of Intensive Care Unit, Sichuan Provincial People’s Hospital, School of Medicine, University of Electronic Science and Technology of China, Chengdu, China

**Keywords:** primary aldosteronism, hypertension management, unilateral adrenalectomy, predictive model, postoperative outcomes

## Abstract

**Introduction:**

Incomplete clinical success after unilateral adrenalectomy for primary aldosteronism (PA) remains a significant challenge, often characterized by persistent hypertension despite biochemical remission.

**Objective:**

This study aimed to develop and validate a preoperative predictive nomogram to estimate the probability of incomplete clinical success in PA patients undergoing unilateral adrenalectomy.

**Materials and methods:**

A retrospective analysis was conducted on 58 PA patients who underwent adrenalectomy. Independent predictors of non-complete clinical success were identified using multivariate logistic regression. A nomogram was developed based on age, highest systolic blood pressure (SBP), and lateralization index (LI). Model performance was evaluated through the concordance index (C-index), calibration plots, and decision curve analysis, with internal validation performed via bootstrapping (1,000 resamples).

**Results:**

Age (OR 1.117), highest SBP (OR 1.241), and LI (OR 1.044) were independently associated with incomplete clinical success. The nomogram showed strong discriminative ability (C-index: 0.829) and good calibration. Internal validation confirmed its reliability (AUC: 0.844, sensitivity 84.2%, specificity 75.0%).

**Conclusion:**

This nomogram offers a reliable, easy-to-use tool for preoperative risk stratification of PA patients, facilitating personalized postoperative management. External validation in multicenter cohorts is warranted.

## 1 Introduction

Primary aldosteronism (PA) is one of the most common endocrine causes of secondary hypertension and is characterized by autonomous overproduction of aldosterone, typically due to benign adrenal lesions such as aldosterone-producing adenomas (APA) or bilateral adrenal hyperplasia (BAH) (1). This excess aldosterone leads to increased sodium retention, volume expansion, and potassium excretion, ultimately resulting in elevated blood pressure (2). Epidemiological studies indicate that PA accounts for approximately 5–13% of all hypertensive patients and up to 20% in those with resistant hypertension (3, 4). Beyond its hemodynamic effects, chronic hyperaldosteronism independently contributes to adverse cardiovascular outcomes. For instance, left ventricular hypertrophy (LVH) is observed in 20–30% of PA patients, and the incidence of stroke is approximately 8.6%, nearly double that seen in the general hypertensive population (5).

Unilateral adrenalectomy remains the treatment of choice for patients with unilateral PA caused by APA or unilateral adrenal hyperplasia, following biochemical confirmation and adrenal venous sampling (AVS)-guided localization of aldosterone excess (6, 7). However, findings from the international, multicenter Primary Aldosteronism Surgery Outcome (PASO) study revealed that only 37% of patients achieve complete clinical success postoperatively (8). According to PASO criteria, incomplete clinical success encompasses both partial and absent clinical responses. Partial success is defined as a reduction in antihypertensive medication requirements without full normalization of blood pressure, whereas absent success reflects no clinical or biochemical improvement despite surgical correction of aldosterone excess (8). This high prevalence of incomplete success poses a significant clinical concern, especially since residual hypertension may result from coexisting essential hypertension or irreversible cardiovascular remodeling (8).

Accurately identifying patients at risk for suboptimal outcomes is therefore of great clinical importance. It facilitates personalized counseling, avoids unrealistic expectations, and helps guide follow-up intensity and antihypertensive strategies. Prior studies have explored a wide range of preoperative predictors, including age, sex, hypertension duration, aldosterone levels, body mass index, renal function, number of antihypertensive medications, and the lateralization index (LI) (9–13). Several multivariable models and nomograms have been proposed to forecast complete clinical success, incorporating combinations of these parameters (14) (15). However, such models often require complex or less accessible inputs, limiting their routine clinical applicability.

To address this unmet need, we aimed to develop and internally validate a simplified, user−friendly nomogram based on three easily obtainable preoperative variables—age, highest systolic blood pressure, and lateralization index—to predict non−complete clinical success after unilateral adrenalectomy. By focusing on this more common but underexplored outcome, our model seeks to complement existing tools and better support individualized perioperative planning in patients with PA.

However, integrating these diverse parameters into a user-friendly and clinically applicable model remains a major challenge. While complex models may improve predictive accuracy, their use in daily clinical settings is often limited due to difficulties in data collection and interpretation. Therefore, there is a clear unmet need for a streamlined, practical predictive tool based on readily available preoperative variables that can support clinical decision-making, optimize surgical planning, and guide individualized patient care.

## 2 Article materials and methods

### 2.1 Study design

**Figure 1:**
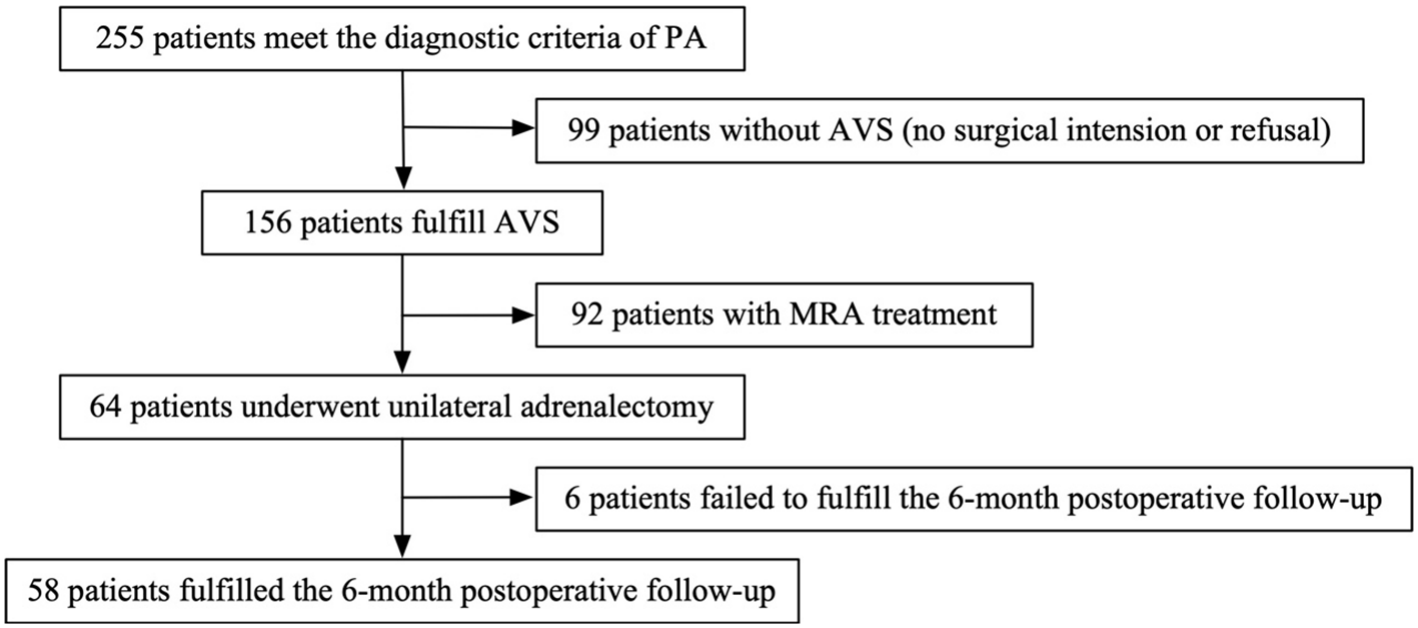
Flowchart of patient selection for inclusion in the analysis.

This retrospective observational study included 255 patients diagnosed with primary aldosteronism (PA) between January 2017 and December 2020 at Sichuan Provincial People’s Hospital, affiliated with the University of Electronic Science and Technology of China. Among them, 58 patients who underwent both adrenal venous sampling (AVS) and subsequent unilateral adrenalectomy were included in the final analysis (**Figure 1**). The study protocol was reviewed and approved by the Ethics Committee of Sichuan Academy of Medical Sciences and Sichuan Provincial People’s Hospital (Ethics Approval No. 1, 2020).

### 2.2 Definition

#### 2.2.1 Diagnosis of primary aldosteronism and unilateral primary aldosteronism

The diagnosis of primary aldosteronism (PA) was made based on the Endocrine Society Clinical Practice Guidelines (7). Prior to biochemical testing, antihypertensive medications were adjusted to minimize interference: α-blockers (e.g., terazosin) and/or non-dihydropyridine calcium channel blockers (e.g., diltiazem) were permitted for at least two weeks, while diuretics were discontinued for a minimum of four weeks. Diagnostic criteria included (1): a positive screening test, defined as a morning orthostatic plasma ARR exceeding 38 (pg/mL/pg/mL); and (2) a positive confirmatory test, characterized by a plasma aldosterone concentration >100 pg/mL following a saline infusion test (SIT) and/or a reduction in plasma aldosterone concentration by less than 30% after a captopril challenge test (CCT).

#### 2.2.2 AVS procedure

Adrenal venous sampling (AVS) was performed by experienced interventional radiologists using a standardized protocol. To ensure hemodynamic stability, patients were positioned supine for at least 30 minutes prior to the procedure. Under sterile conditions, a catheter was introduced percutaneously via the femoral vein and advanced into the inferior vena cava, followed by selective cannulation of both the left and right adrenal veins. Contrast media were used to verify accurate catheter placement. Sequential blood samples were collected from the bilateral adrenal veins and the inferior vena cava for subsequent aldosterone and cortisol measurements. All procedures followed a non-cosyntropin-stimulated protocol to preserve baseline hormonal levels and allow for physiologic assessment of lateralization.

#### 2.2.3 Interpretation and cut-off points of the AVS indices

AVS was performed under fluoroscopic guidance with blood samples collected from the left and right adrenal veins and the inferior vena cava. Hormone concentrations were measured using chemiluminescence immunoassays, with intra-assay and inter-assay coefficients of variation maintained below 5%. Selectivity index (SI) was used to assess successful adrenal vein cannulation, defined as the cortisol concentration ratio between the adrenal vein and inferior vena cava (SI ≥ 2). Only samples meeting this criterion were considered valid for further interpretation. To determine lateralization of aldosterone secretion, the lateralization index (LI) was calculated as the aldosterone-to-cortisol ratio of the dominant adrenal vein divided by that of the nondominant side. An LI ≥ 2 was considered indicative of unilateral aldosterone hypersecretion, while an LI < 2 suggested bilateral disease. Additional indices, such as the contralateral suppression index and the aldosterone-to-cortisol ratio of the nondominant vein relative to the inferior vena cava, were also evaluated to enhance diagnostic confidence.

#### 2.2.4 Criteria of unilateral adrenalectomy outcomes

Postoperative outcomes were evaluated in patients with unilateral PA who completed at least six months of follow-up after adrenalectomy. Both clinical and biochemical outcomes were assessed according to the internationally recognized Primary Aldosteronism Surgical Outcome (PASO) criteria (8). According to PASO definitions, incomplete clinical success includes both: 1) Partial success, defined as a reduction in the number or dosage of antihypertensive medications without full normalization of blood pressure; Absent success, indicating no meaningful improvement in clinical or biochemical parameters.

#### 2.2.5 Definition of highest systolic blood pressure

As part of the clinical data collection, the highest recorded systolic blood pressure (SBP) prior to adrenalectomy was extracted from outpatient or inpatient medical records. This value was defined as the maximum SBP measured during any clinical encounter within three months prior to surgery, using validated automated sphygmomanometers. In patients with multiple readings, the peak SBP value was selected to reflect the maximum hemodynamic burden before intervention.

This variable was included as a predictor in the nomogram due to its clinical relevance as a marker of long-term vascular load and potential irreversible arterial remodeling.

#### 2.2.6 Analytical methods

Blood samples were collected in EDTA tubes, immediately centrifuged at 1500 g for 10 minutes at 4°C, and the plasma was stored at −80°C until analysis. Plasma aldosterone concentrations (pg/mL) were measured using a chemiluminescence assay (Antu Biotech Co., Ltd, Zhengzhou, China) with an assay sensitivity of 10 pg/mL. The intra- and inter-assay coefficients of variation ranged from 2.502% to 3.304% and from 0.955% to 2.918%, respectively. Direct renin concentrations (pg/mL) were also determined using a chemiluminescence assay from the same manufacturer, with an assay sensitivity of 0.5 pg/mL and intra- and inter-assay coefficients of variation ranging from 1.818% to 1.830% and from 4.751% to 6.461%, respectively. Serum cortisol (µg/L) was quantified using a competitive electrochemiluminescence immunoassay on a Cobas e801 analyzer (Roche Diagnostics GmbH, Germany), with an analytical sensitivity of ≤0.109 µg/dL (3.0 nmol/L). The intra- and inter-assay coefficients of variation for cortisol measurements were 1.1% to 5.5% and 1.8% to 7.3%, respectively. All other biochemical parameters were analyzed in plasma or serum using standardized laboratory methods.

#### 2.2.7 Development and assessment of the nomogram

A multivariate logistic regression model was constructed to predict incomplete clinical success following unilateral adrenalectomy in patients with primary aldosteronism (PA). Candidate variables were first screened using univariate logistic regression (significance threshold: P < 0.10), and significant predictors were subsequently selected via stepwise multivariate regression (P < 0.05). The final set of independent predictors—age, highest systolic blood pressure (SBP), and lateralization index (LI)—were incorporated into a nomogram to estimate the individualized probability of incomplete clinical success. Each predictor was assigned a weighted point value based on its relative contribution to the model. Points were summed to generate a total score, which was then projected onto a probability scale to derive the predicted risk. Model performance was evaluated through several key metrics: 1) Calibration was assessed by plotting predicted probabilities against observed outcomes; 2) Discrimination was quantified using the area under the receiver operating characteristic curve (AUC), the concordance index (C-index), and the discrimination slope; 3) Goodness-of-fit was assessed using the Hosmer–Lemeshow test and the coefficient of determination (R²); 4) Clinical utility was assessed via decision curve analysis (DCA) across a range of probability thresholds to determine net benefit. To ensure robustness and internal validity, the nomogram was subjected to bootstrap resampling (1,000 iterations), which confirmed its reproducibility and predictive accuracy.

#### 2.2.8 Predictive Accuracy and Net Clinical Benefit of the Nomogram

**Figure 2:**
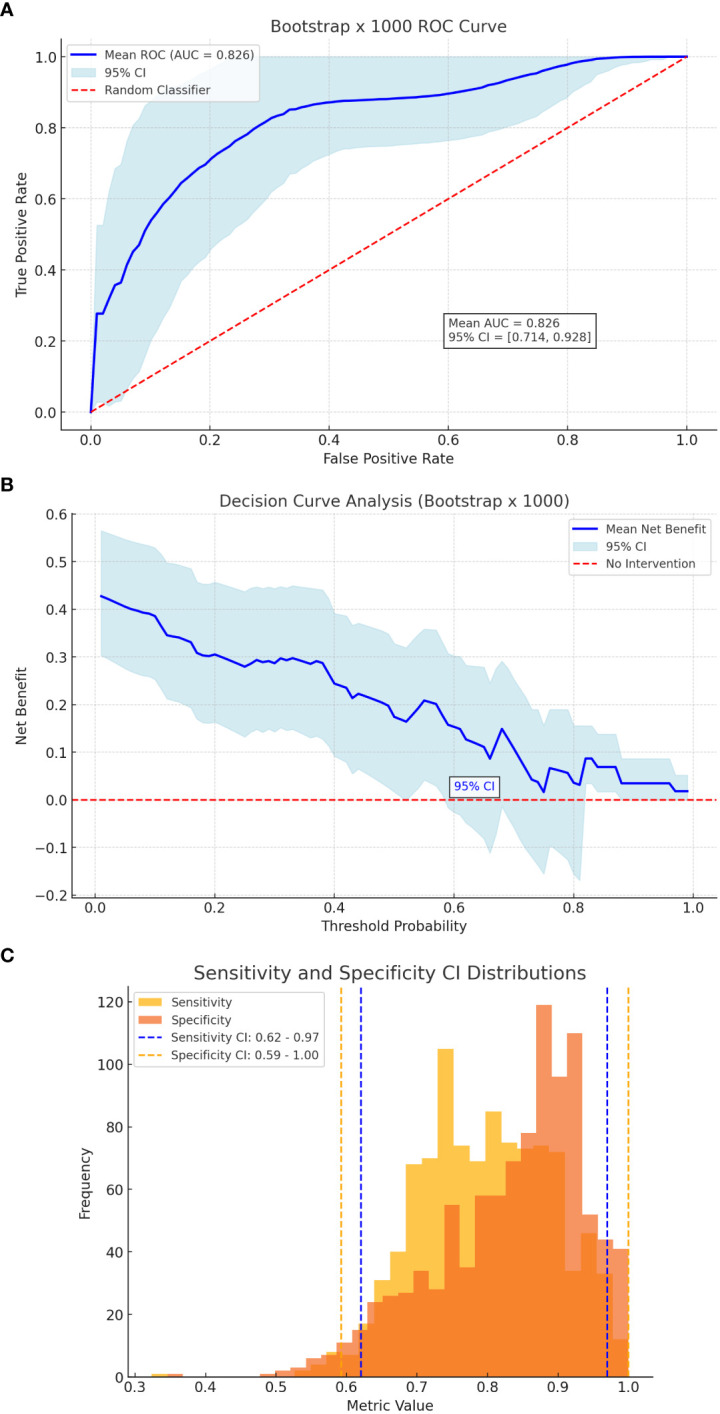
Bootstrap Validation. **(A)** ROC curve of bootstrap. **(B)** Decision curve analysis of bootstrap. **(C)** Sensitivity & specificity CI distributions.

Internal validation of the nomogram was conducted using bootstrap resampling with 1,000 iterations to evaluate its stability and generalizability. The model achieved a mean area under the ROC curve (AUC) of 0.844 (95% CI: 0.650–0.911), indicating excellent discriminative ability (**Figure 2A**). Using Youden’s index as the threshold criterion, the model demonstrated an optimal sensitivity of 84.2% and specificity of 75.0%. The reliability of these estimates was supported by bootstrap-derived confidence intervals, with sensitivity ranging from 62% to 97% and specificity from 59% to 100% (**Figure 2C**). Decision curve analysis (DCA) confirmed that the nomogram provides a consistent net clinical benefit across a broad spectrum of threshold probabilities, reinforcing its clinical applicability in distinguishing between patients at high versus low risk of incomplete clinical success. Collectively, these findings support the use of the nomogram as a robust and practical tool for individualized risk stratification and evidence-based decision-making in the preoperative management of patients with primary aldosteronism.

#### 2.2.9 Statistical analysis

All available data were included in the final analysis. Statistical computations were conducted using R software (version 4.0.3) and Python (version 3.9). R was primarily employed for nomogram construction and visualization, utilizing packages such as rms, pROC, ggplot2, and dca. Python, with libraries including scikit-learn and matplotlib, was used to perform logistic regression modeling, calibration curve plotting, and receiver operating characteristic (ROC) analysis. Continuous variables were expressed as mean ± standard deviation (SD) if normally distributed, or as median with interquartile range (IQR: P25–P75) if non-normally distributed. Categorical variables were summarized as frequencies and percentages (%). All statistical tests were two-sided, and a P value < 0.05 was considered statistically significant.

#### 2.2.10 Disclosures

This study was supported by Key R&D Program of Sichuan Science and Technology Plan in 2023 Project No. 23ZDYF1960. All authors had full access to all the data in the study and accept responsibility to submit for publication.

## 3 Results

### 3.1 General characteristics

A total of 255 patients diagnosed with primary aldosteronism (PA) were screened for inclusion. Among them, 99 patients did not undergo adrenal venous sampling (AVS) due to lack of surgical intent or personal refusal. Of the remaining 156 patients who completed AVS, 92 were treated non-surgically with mineralocorticoid receptor antagonists (MRAs). Ultimately, 64 patients underwent unilateral adrenalectomy, of whom 58 completed the 6-month postoperative follow-up and were included in the final analysis (**Figure 1**). Based on the PASO criteria, 25 patients (43.1%) were classified as having incomplete clinical success, and 33 patients (56.9%) achieved complete clinical success. The baseline clinical, biochemical, and imaging characteristics of the two groups are summarized in **Table 1**.

**Table 1:** Baseline characteristics in the training cohort.

Variable	Incomplete clinical success	Complete clinical success	P value
Number	25	33	
Age	57(44–65)	46(39–54)	0.013
Gender (male, %)	13(52)	11(33.3)	0.156
Body mass index (kg/m^2^)	25.8(22.7-29.7)	23.5(21.5-25.2)	0.017
Hypertension (mmHg)			
History of hypertension	24(96)	32(97)	0.842
Duration of hypertension, y	10(1-12)	4(2-10)	0.321
Highest SBP	183(180-200)	170(163-180)	0.002
Highest DBP	100(100-110)	101(100-110)	0.923
SBP at admission	155(148-166)	160(150-167)	0.446
DBP at admission	99(88-104)	97(89-105)	0.814
Hypokalemia (mmol/L)			
History of hypokalemia	13(52)	21(63.6)	0.377
Duration of hypokalemia	0.02(0.0-0.17)	0.08(0.0-1.0)	0.173
Nadir Potassium Level	3.0(2.61-3.49)	2.48(2.13-3.19)	0.023
Potassium Level at admission	3.13(2.94-3.53)	3.0(2.83-3.28)	0.242
eGFR (ml/min^-1^ per 1.73m^2^)	95(82-109)	107(102-119)	0.030
UACR	38(17-57)	43(22-67)	0.258
PAC (ng/dl^-1^)	323.9(198.2-564.0)	336.0(251.6-654.9)	0.677
DRC (ng dl^-1^n^-1^)	2.31(1.52-3.3)	2.10(0.87-2.88)	0.451
ARR	109.5(72.1-389.1)	164.8(82.8-546.2)	0.505
LI	5.96(3.84-19.46)	9.40(3.90-16.47)	0.721
CSI	0.43(0.31-0.66)	0.26(0.15-0.52)	0.191
Typical imaging feature	19(76)	30(90.9)	0.124
Size of largest nodule, mm	10.0(7.75-15)	15.0(10.0-20.75)	0.057

SBP, Systolic Blood Pressure; DBP, Diastolic Blood Pressure; eGFR, Estimated Glomerular Filtration Rate;Urine Albumin-to-Creatinine Ratio; PAC, Plasma Aldosterone Concentration; DRC, Direct Renin Concentration; ARR, Aldosterone-to-Renin Ratio; LI, lateralization index; CSI, contralateral suppression index;

Data shown as median (interquartile range) or number (%).

Hypokalaemia: defined as serum potassium concentration of ≤3.5meql^-1^ or requirement of potassium supplementation.

Patients with incomplete clinical success were significantly older, with a median age of 57 years (IQR 44–65), compared to 46 years (IQR 39–54) in the complete success group (P = 0.013). They also had a higher body mass index (BMI) [25.8 kg/m² (IQR 22.7–29.7) vs. 23.5 kg/m² (IQR 21.5–25.2), P = 0.017], and a higher maximum systolic blood pressure (SBP) [183 mmHg (IQR 180–200) vs. 170 mmHg (IQR 163–180), P = 0.002]. In addition, the nadir potassium level was elevated in the incomplete success group [3.0 mmol/L (IQR 2.61–3.49) vs. 2.48 mmol/L (IQR 2.13–3.19), P = 0.023], while their estimated glomerular filtration rate (eGFR) was lower [95 mL/min/1.73 m² (IQR 82–109) vs. 107 mL/min/1.73 m² (IQR 102–119), P = 0.030].

No significant differences were observed in the lateralization index (LI) [8.6 vs. 9.3, P = 0.530] or the size of the largest adrenal nodule [2.1 cm vs. 2.2 cm, P = 0.839] between the two groups.

### 3.2 Univariate and multivariate logistic regression analysis

In the univariate logistic regression analysis, several variables were significantly associated with incomplete clinical success (**Table 2**). These included: age (odds ratio [OR], 1.061; 95% confidence interval [CI], 1.013–1.110; P = 0.015), body mass index (BMI) (OR, 1.240; 95% CI, 1.051–1.464; P = 0.013), highest systolic blood pressure (SBP) (OR, 1.060; 95% CI, 1.020–1.110; P = 0.004), nadir potassium level (OR, 2.960; 95% CI, 1.202–7.290; P = 0.018), and estimated glomerular filtration rate (eGFR) (OR, 0.950; 95% CI, 0.905–0.995; P = 0.031) (**Table 2**). Although the lateralization index (LI) was not statistically significant in the univariate analysis (OR = 1.014, 95% CI: 0.970–1.059, P = 0.547), it was retained in the multivariate model due to its known clinical relevance in the context of primary aldosteronism. Subsequent stepwise multivariate logistic regression identified three independent predictors of incomplete clinical success (**Table 3**): highest SBP (OR, 1.241; 95% CI, 1.082–1.425; P = 0.003), LI (OR, 1.044; 95% CI, 1.008–1.082; P = 0.018), and age (OR, 1.117; 95% CI, 1.012–1.234; P = 0.029) (**Table 3**).

**Table 2:** Factors associated with non-complete clinical success after adrenalectomy on univariate logistic regression analysis.

Variable	OR	95%CI	P
Age	1.06	1.01-1.11	0.015
BMI, kg/m^2^	1.24	1.05-1.47	0.013
Highest SBP (mmHg)	1.06	1.02-1.11	0.004
Nadir Potassium Level	2.96	1.2-7.3	0.018
eGFR (ml/min^-1^ per 1.73m^2^)	0.98	0.95-1.00	0.083
LI	1.00	0.99-1.01	0.835
Size of largest nodule, mm	0.91	0.84-1.00	0.050

**Table 3:** Stepwise selection(forward) of predictors of non-complete clinical success.

Position in model	Variable	OR (95%CI)	P
First	Highest SBP(mmHg)	1.24(1.08-1.43)	0.003
Second	LI	1.04(1.01-1.07)	0.018
Third	Age	1.12(1.01-1.24)	0.029

### 3.3 Risk prediction nomogram development and validation

**Figure 3:**
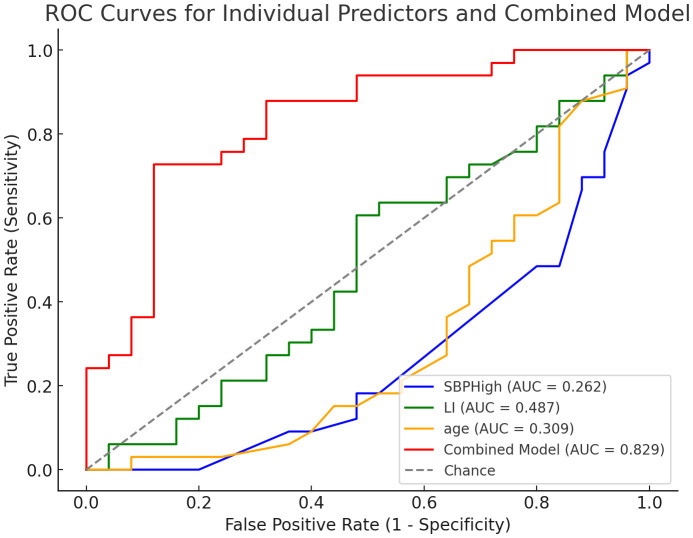
Receiver operating characteristic (ROC) curve for the predictive model. C-index is 0.829.

A logistic regression model was developed using three independent predictors identified through multivariate analysis: highest systolic blood pressure (SBP), lateralization index (LI), and age (**Table 3**). The combined model demonstrated excellent discriminatory performance, with an area under the ROC curve (AUC) of 0.829 (**Figure 3**). At the optimal cutoff point determined by Youden’s index, the model achieved a sensitivity of 84.2% and specificity of 75.0%.

**Figure 4:**
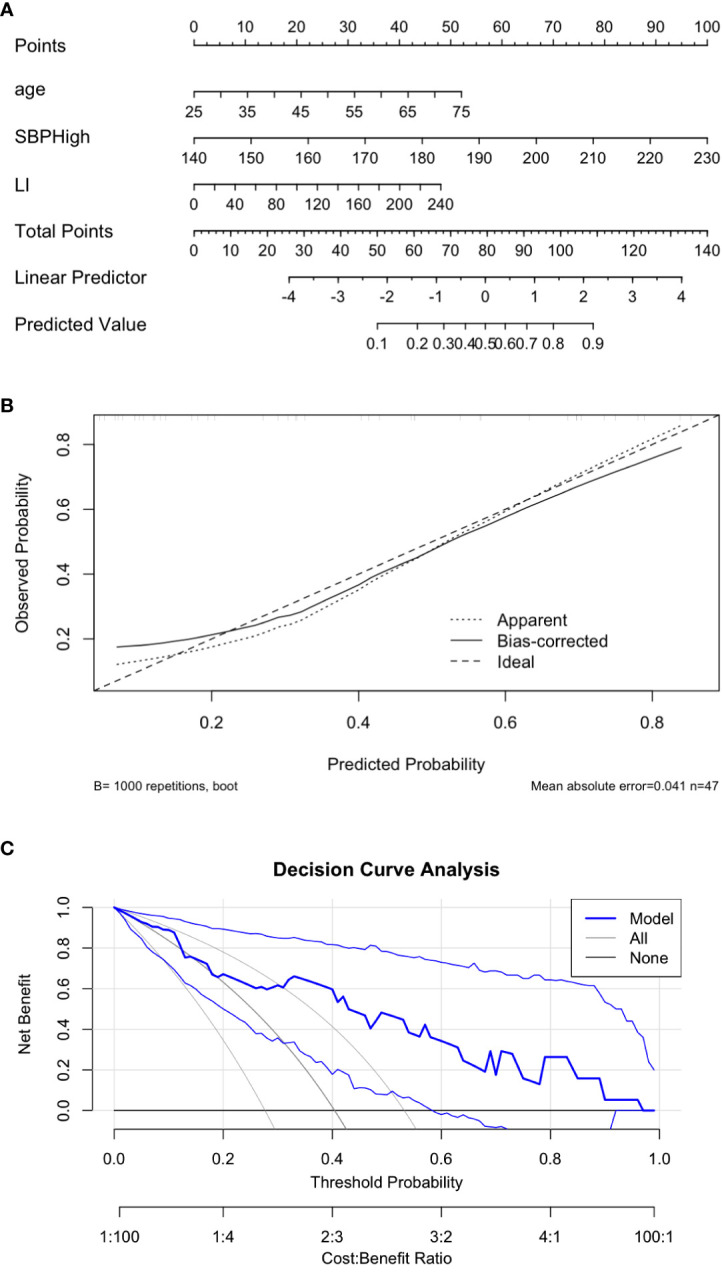
Visualization and performance of the predictive nomogram. **(A)** Nomogram predicting the probability of incomplete clinical success. C-index 0.829. **(B)** Calibration plot comparing predicted and observed outcomes. **(C)** Decision curve analysis demonstrating clinical utility.

A nomogram incorporating these three predictors was constructed to facilitate individualized risk prediction (**Figure 4A**). The nomogram showed high accuracy in estimating the probability of incomplete clinical success, with a bootstrap-corrected C-index of 0.829. The calibration plot indicated strong agreement between predicted probabilities and observed outcomes (**Figure 4B**), and decision curve analysis (DCA) confirmed consistent net clinical benefit across a wide range of threshold probabilities (**Figure 4C**), underscoring the nomogram’s clinical utility.

The Hosmer–Lemeshow test indicated good model calibration (P = 0.574), suggesting that the model fit the observed data well.

To further assess model robustness and generalizability, bootstrap resampling (1,000 iterations) was performed. The internal validation yielded a mean AUC of 0.844 (95% CI: 0.650–0.911) (**Figure 2A**). Sensitivity and specificity remained high across the resampling iterations, with 95% confidence intervals ranging from 62% to 97% for sensitivity and 59% to 100% for specificity (**Figure 2C**).

DCA conducted on the bootstrapped data reaffirmed the model’s clinical benefit, particularly in distinguishing patients at higher vs. lower risk of incomplete clinical success (**Figure 2B**). These findings collectively support the nomogram’s use as a practical, evidence-based tool to aid preoperative decision-making in patients with primary aldosteronism.

## 4 Discussion

In this retrospective study, we developed and internally validated a nomogram to predict the risk of incomplete clinical success following unilateral adrenalectomy in patients with primary aldosteronism. The final predictive model incorporated three preoperative variables—highest systolic blood pressure, lateralization index, and age—which were independently associated with postoperative outcomes. The nomogram demonstrated strong discriminative ability (AUC = 0.829; bootstrap-corrected AUC = 0.844) and good calibration, with consistent net clinical benefit confirmed by decision curve analysis. These findings indicate that our model may serve as a practical tool for preoperative risk stratification, enabling clinicians to identify patients less likely to achieve complete clinical success after surgery and to plan for enhanced postoperative monitoring or adjunctive therapy.

Several prior studies have explored predictors of postoperative outcomes in patients with primary aldosteronism undergoing adrenalectomy. Variables such as age, duration of hypertension, aldosterone-to-renin ratio, body mass index, and the presence of target organ damage have been previously reported as significant determinants of clinical success (15–19). Notably, the PASO study, a large international multicenter cohort, demonstrated that younger age, shorter hypertension duration, and absence of comorbidities were associated with higher rates of complete clinical success (8). Our findings are partially consistent with these reports. We confirmed age as an independent predictor, supporting the hypothesis that younger patients may exhibit more reversible vascular and renal changes. Unlike earlier models that incorporated numerous variables—some of which may not be readily accessible or consistently measured in routine clinical settings—our model utilizes only three objective, preoperative parameters: age, SBP, and LI. This simplified structure enhances clinical applicability without compromising predictive performance, as reflected in the high AUC and favorable calibration. Although LI did not reach statistical significance in the univariate analysis, it became a significant predictor in multivariate analysis, reaffirming its value in surgical planning, as also shown in studies such as Tagawa et al (14). Compared to existing postoperative prediction models—such as those by Zhang et al. (2017), Yang et al. (2020), and Tagawa et al. (2017) which primarily focused on complete clinical success, our study offers a complementary perspective by addressing non-complete clinical success (14, 15, 20). This outcome is more prevalent and clinically meaningful, especially for patients with residual hypertension despite biochemical cure. Existing models often include five or more predictors or rely on subjective or invasive inputs, which limits practicality. In contrast, our nomogram provides a streamlined and accessible tool suitable for broader clinical implementation.

The prognostic value of the variables in our model is supported by their underlying pathophysiological roles. Elevated systolic blood pressure prior to adrenalectomy likely reflects the cumulative burden of long-standing hypertension, which may induce irreversible structural changes such as arterial stiffening, vascular remodeling, and left ventricular hypertrophy (21–23). These alterations can persist even after biochemical correction of aldosterone excess, thereby limiting postoperative blood pressure normalization. Similarly, increased age has been consistently associated with poorer clinical outcomes in PA, potentially due to age-related decline in vascular elasticity, renal function, and autonomic regulation, all of which contribute to fixed hypertension phenotypes less responsive to surgical correction. Moreover, older patients may have a higher likelihood of coexisting essential hypertension, further diminishing the potential for complete clinical success. The lateralization index, a measure of asymmetry in aldosterone production, reflects the degree of unilateral dominance and guides surgical eligibility. A higher LI typically indicates more discrete, localized aldosterone overproduction, which is more amenable to resolution via unilateral adrenalectomy (24, 25). Thus, patients with lower LI values may have more diffuse or bilateral adrenal involvement, reducing the likelihood of full clinical remission even with technically successful surgery. In our center, adrenal venous sampling (AVS) was performed without cosyntropin stimulation. Based on institutional experience and internal validation, we adopted a lateralization index threshold of >2 to define unilateral aldosterone excess. Although international guidelines such as the 2016 Endocrine Society Clinical Practice Guideline recommend a more conservative cut-off of LI ≥4 when cosyntropin is used, several studies have demonstrated that an LI >2 without cosyntropin still predicts favorable surgical outcomes. This reflects real-world variability in AVS protocols and emphasizes the importance of considering additional supportive evidence, such as contralateral suppression or imaging concordance. Nevertheless, we acknowledge that this choice may limit the generalizability of our model to centers using stricter criteria and have now clarified this in the discussion.

These pathophysiological insights further support the utility of our nomogram, which provides a simple yet effective tool for individualized preoperative risk assessment. By incorporating only three readily accessible clinical variables—age, highest systolic blood pressure, and lateralization index—the model enables clinicians to estimate the individualized probability of incomplete clinical success with minimal additional burden. This tool may be particularly valuable in guiding shared decision-making, especially in borderline cases where the anticipated surgical benefit is uncertain. Patients identified as having a high predicted risk of incomplete response may benefit from more conservative surgical counseling, closer postoperative follow-up, or early initiation of adjunctive medical therapy, such as mineralocorticoid receptor antagonists. Finally, the nomogram’s simplicity and strong internal validation make it well-suited for integration into clinical workflows, including electronic health record (EHR) systems and mobile decision-support applications. Its ability to aid personalized risk stratification may also improve resource allocation by prioritizing patients most likely to benefit from surgical intervention.

Several limitations of this study should be acknowledged. First, the study was conducted at a single tertiary referral center with a relatively small sample size (n = 58), which may limit the generalizability of the findings. Although internal validation using bootstrap resampling enhanced the robustness of the model, external validation in independent multicenter cohorts is necessary before widespread clinical adoption. Second, the retrospective nature of the study may introduce selection bias and information bias, particularly in the extraction of clinical and blood pressure data from medical records. While efforts were made to standardize data collection, the influence of undocumented variables, such as medication adherence or lifestyle factors, cannot be excluded. In particular, the highest preoperative systolic blood pressure value, though clinically meaningful, was extracted from routine outpatient or inpatient records and may be susceptible to situational influences such as the white coat effect, potentially introducing variability not entirely reflective of the patient’s usual hemodynamic burden. Third, the study focused exclusively on clinical outcomes (blood pressure response) and did not evaluate biochemical success or long-term cardiovascular endpoints, which are also clinically relevant in primary aldosteronism. Finally, although the nomogram is based on simple clinical parameters, its predictive performance may vary in populations with different ethnic backgrounds, comorbidity profiles, or diagnostic protocols. Future research should address these factors through prospective validation and integration into real-world clinical settings. It is important to note that while our model predicts the likelihood of incomplete clinical success, it is not intended to guide the initial choice between surgical and medical therapy, which should be based on broader evidence including long-term cardiovascular outcomes.

## 5 Conclusion

In summary, we developed and internally validated a concise, clinically applicable nomogram to predict the risk of incomplete clinical success following unilateral adrenalectomy in patients with primary aldosteronism. By integrating three readily available preoperative variables—age, highest systolic blood pressure, and lateralization index—the model demonstrated excellent discriminative performance and calibration. This tool may support individualized preoperative counseling, optimize clinical decision-making, and guide postoperative management strategies. External validation in prospective, multicenter cohorts is warranted to confirm its generalizability and real-world clinical utility.

## Data Availability

The original contributions presented in the study are included in the article/supplementary material. Further inquiries can be directed to the corresponding author.

## References

[1] Stowasser M. Update in primary aldosteronism. J Clin Endocrinol Metab. (2015) 100:1–10. doi: 10.1210/jc.2014-3663, PMID: 25365316

[2] Rossi GPD. Diagnosis and treatment of primary aldosteronism. Rev Endocr Metab Disord. (2011) 12:27–36. doi: 10.1007/s11154-011-9162-8, PMID: 21369868

[3] Calhoun DA Nishizaka MK Zaman MA Thakkar RB Weissmann P. Hyperaldosteronism among black and white subjects with resistant hypertension. Hypertension. (2002) 40:892–6. doi: 10.1161/01.hyp.0000040261.30455.b6, PMID: 12468575

[4] Rossi GP Bernini G Caliumi C Desideri G Fabris B Ferri C. A prospective study of the prevalence of primary aldosteronism in 1,125 hypertensive patients. J Am Coll Cardiol. (2006) 48:2293–300. doi: 10.1016/j.jacc.2006.07.059, PMID: 17161262

[5] Prejbisz A Warchoł-Celińska E Lenders JWM Januszewicz A. Cardiovascular risk in primary hyperaldosteronism. Horm Metab Res. (2015) 47:973–80. doi: 10.1055/s-0035-1565124, PMID: 26575306

[6] Young WF. Diagnosis and treatment of primary aldosteronism: practical clinical perspectives. J Intern Med. (2019) 285:126–48. doi: 10.1111/joim.12831, PMID: 30255616

[7] Funder JW Carey RM Mantero F Murad MH Reincke M Shibata H. The management of primary aldosteronism: case detection, diagnosis, and treatment: an endocrine society clinical practice guideline. J Clin Endocrinol Metab. (2016) 101:1889–916. doi: 10.1210/jc.2015-4061, PMID: 26934393

[8] Williams TA Lenders JWM Mulatero P Burrello J Rottenkolber M Adolf C. Outcomes after adrenalectomy for unilateral primary aldosteronism: an international consensus on outcome measures and analysis of remission rates in an international cohort. Lancet Diabetes Endocrinol. (2017) 5:689–99. doi: 10.1016/S2213-8587(17)30135-3, PMID: 28576687 PMC5572673

[9] Sawka AM Young WF Thompson GB Grant CS Farley DR Leibson C. Primary aldosteronism: factors associated with normalization of blood pressure after surgery. Ann Intern Med. (2001) 135:258–61. doi: 10.7326/0003-4819-135-4-200108210-00010, PMID: 11511140

[10] Wachtel H Cerullo I Bartlett EK Kelz RR Cohen DL Karakousis GC. Long-term blood pressure control in patients undergoing adrenalectomy for primary hyperaldosteronism. Surgery. (2014) 156:1394–402. doi: 10.1016/j.surg.2014.08.021, PMID: 25456918

[11] Zhang X Zhu Z Xu T Shen Z. Factors affecting complete hypertension cure after adrenalectomy for aldosterone-producing adenoma: outcomes in a large series. Urol Int. (2013) 90:430–4. doi: 10.1159/000347028, PMID: 23466491

[12] Worth PJ Kunio NR Siegfried I Sheppard BC Gilbert EW. Characteristics predicting clinical improvement and cure following laparoscopic adrenalectomy for primary aldosteronism in a large cohort. Am J Surg. (2015) 210:702–9. doi: 10.1016/j.amjsurg.2015.05.033, PMID: 26323999

[13] Murashima M Trerotola SO Fraker DL Han D Townsend RR Cohen DL. Adrenal venous sampling for primary aldosteronism and clinical outcomes after unilateral adrenalectomy: a single-center experience. J Clin Hypertens (Greenwich). (2009) 11:316–23. doi: 10.1111/j.1751-7176.2009.00120.x, PMID: 19527322 PMC8673066

[14] Tagawa M Ghosn M Wachtel H. Lateralization index but not contralateral suppression at adrenal vein sampling predicts improvement in blood pressure after adrenalectomy for primary aldosteronism. J Hum Hypertension. (2017) 31:444–449. doi: 10.1038/jhh.2016.92, PMID: 28079049

[15] Yang Y Williams TA Song Y Yang S He W Wang K. Nomogram-based preoperative score for predicting clinical outcome in unilateral primary aldosteronism. J Clin Endocrinol Metab. (2020) 105:e4382–92. doi: 10.1210/clinem/dgaa634, PMID: 32898224

[16] Park JB Schiffrin EL. Small artery remodeling is the most prevalent (earliest)? form of target organ damage in mild essential hypertension. J Hypertens. (2001) 19:921–30. doi: 10.1097/00004872-200105000-00013, PMID: 11393676

[17] Küpers EM Amar L Raynaud A Plouin P-F Steichen O. A clinical prediction score to diagnose unilateral primary aldosteronism. . J Clin Endocrinol Metab. (2012) 97:3530–7. doi: 10.1210/jc.2012-1917, PMID: 22918872

[18] Huang C-W Lee B-C Liu K-L Chang Y-C Wu V-C Lee P-T. Preoperative non-stimulated adrenal venous sampling index for predicting outcomes of adrenalectomy for unilateral primary aldosteronism. J Formos Med Assoc. (2020) 119:1185–92. doi: 10.1016/j.jfma.2020.04.016, PMID: 32386674

[19] Hannon MJ Sze WC Carpenter R Parvanta L Matson M Sahdev A. Clinical outcomes following unilateral adrenalectomy in patients with primary aldosteronism. QJM. (2017) 110:277–81. doi: 10.1093/qjmed/hcw194, PMID: 28180906

[20] Zhang Y Niu W Zheng F Zhang H Zhou W Shen Z. Identifying unilateral disease in Chinese patients with primary aldosteronism by using a modified prediction score. J Hypertension. (2017) 35:2486–92. doi: 10.1097/HJH.0000000000001488, PMID: 28708774 PMC5673302

[21] Dominguez DA Chatani P Murphy R Copeland AR Chang R Sadowski SM. Contralateral suppression index does not predict clinical cure in patients undergoing surgery for primary aldosteronism. Ann Surg Oncol. (2021) 28:7487–95. doi: 10.1245/s10434-021-09692-7, PMID: 33939050 PMC8530859

[22] Umakoshi H Tsuiki M Yokomoto-Umakoshi M Takeda Y Takashi Y Kurihara I. Correlation between lateralization index of adrenal venous sampling and standardized outcome in primary aldosteronism. J Endocr Soc. (2018) 2:893–902. doi: 10.1210/js.2018-00055, PMID: 30057970 PMC6057509

[23] Li S Sun H Ma L Zhu Y Xie W Sun J. Left-versus-right-adrenal-volume ratio as a screening index before adrenal venous sampling to identify unilateral primary aldosteronism patients. J Hypertens. (2020) 38:347–53. doi: 10.1097/HJH.0000000000002271, PMID: 31584510

[24] Suntornlohanakul O Soonthornpun S Srisintorn W Murray RD Kietsiriroje N. Performance of the unilateral AV/IVC index in primary hyperaldosteronism subtype prediction: A validation study in a single tertiary centre. Clin Endocrinol (Oxf). (2020) 93:111–8. doi: 10.1111/cen.14210, PMID: 32347973

[25] Cho EH. Update on the aldosterone resolution score and lateralization in patients with primary aldosteronism. Endocrinol Metab (Seoul). (2018) 33:352–4. doi: 10.3803/EnM.2018.33.3.352, PMID: 30229574 PMC6145955

